# Brain regions involved in processing facial identity and expression are differentially selective for surface and edge information

**DOI:** 10.1016/j.neuroimage.2014.04.032

**Published:** 2014-08-15

**Authors:** Richard J. Harris, Andrew W. Young, Timothy J. Andrews

**Affiliations:** Department of Psychology and York Neuroimaging Centre, University of York, York YO10 5DD, United Kingdom

**Keywords:** Vision, Face, Identity, Expression

## Abstract

Although different brain regions are widely considered to be involved in the recognition of facial identity and expression, it remains unclear how these regions process different properties of the visual image. Here, we ask how surface-based reflectance information and edge-based shape cues contribute to the perception and neural representation of facial identity and expression. Contrast-reversal was used to generate images in which normal contrast relationships across the surface of the image were disrupted, but edge information was preserved. In a behavioural experiment, contrast-reversal significantly attenuated judgements of facial identity, but only had a marginal effect on judgements of expression. An fMR-adaptation paradigm was then used to ask how brain regions involved in the processing of identity and expression responded to blocks comprising all normal, all contrast-reversed, or a mixture of normal and contrast-reversed faces. Adaptation in the posterior superior temporal sulcus – a region directly linked with processing facial expression – was relatively unaffected by mixing normal with contrast-reversed faces. In contrast, the response of the fusiform face area – a region linked with processing facial identity – was significantly affected by contrast-reversal. These results offer a new perspective on the reasons underlying the neural segregation of facial identity and expression in which brain regions involved in processing invariant aspects of faces, such as identity, are very sensitive to surface-based cues, whereas regions involved in processing changes in faces, such as expression, are relatively dependent on edge-based cues.

## Highlights

• Different brain regions are involved in recognition of facial identity and expression.
• Unclear how these regions process different visual properties from the face.
• This issue was addressed using contrast reversal of faces and fMR-adaptation.
• Brain regions involved in processing identity are sensitive to surface-based cues.
• Regions involved in processing expression are dependent on edge-based cues.

## Introduction

Models of human face perception suggest that facial identity and expression are processed along two different neural pathways (Bruce and Young, 1986, Bruce and Young, 2012, Haxby et al., 2000). Support for the idea of separable pathways in face perception comes from neuroimaging studies that have investigated the selectivity of face regions in the human brain (Andrews and Ewbank, 2004, Hoffman and Haxby, 2000, Winston et al., 2004). A posterior part of the superior temporal sulcus (pSTS) is thought to be important in processing movements of the face, such as changes in gaze and expression, which are important for social interactions (Baseler et al., 2013, Engell and Haxby, 2007, Harris et al., 2012, Psalta et al., 2013). In contrast, a region in the fusiform gyrus, the fusiform face area (FFA), is considered to be important for the representation of facial characteristics that are important for recognition (Davies-Thompson et al., 2013, Grill-Spector et al., 2004, Rotshtein et al., 2005).

Central to understanding this neural segregation of analyses of identity and expression is the question of the extent to which it may be driven by visual properties of faces themselves (Calder and Young, 2005). Bruce and Young (1998) drew attention to the fact that any facial image consists of a set of edges created by abrupt changes in reflectance that define the shapes and positions of facial features and a broader pattern of surface pigmentation resulting from local changes in the reflectance properties of the skin. These properties of shape and pigmentation may contribute differentially to the perception of identity and expression. Bruce and Young (1998) suggested that feature shapes (i.e. edge-based information) may be critical for perceiving facial expressions, with surface pigmentation being relatively important to identity.

A useful way of testing the importance of edge- and surface-based cues in face perception is with contrast reversal (as in a photo negative). In a contrast-reversed image the edges that define feature shapes remain in the same positions, despite the huge change in overall surface properties. A variety of evidence shows that facial expressions can still be recognised in contrast-reversed images (Bruce and Young, 1998, Magnussen et al., 1994, White, 2001). Recognition of facial identity, however, is severely disrupted by contrast-reversal, showing the importance of surface patterns to the recognition of facial identity (Bruce and Langton, 1994, Burton et al., 2005, Russell et al., 2006). Although high spatial frequency, edge-based information also makes an important contribution to the perception of identity (Burton et al., 2005, Fiorentini et al., 1983, Goffaux et al., 2005), it does not support recognition on its own. For example, line drawings of faces are not usually sufficient for the accurate recognition or discrimination of identity (Davies et al., 1978, Leder, 1999) unless they are caricatured (Rhodes and Tremewan, 1994) or given some limited textural information by ‘thresholding’ the original image (Bruce et al., 1992).

A broad distinction, then, can be made between the visual information that is important for different aspects of face perception. For the perception of facial identity, contrast patterns and edge-based shape cues can both convey useful information. However, the perception of facial expression is relatively dependent on edge-based, shape cues that correlate with movements of the facial muscles, and less dependent on textural contrast patterns. Here, we introduce a striking demonstration of this reliance of facial expression perception on shape information rather than contrast patterns by showing that, behaviourally, facial expression perception is insensitive to contrast-reversal to the point where it is not difficult to match expressions across normal and contrast-reversed images, despite the large differences between the images. As expected, however, identity perception is markedly impaired under the same conditions. We then used this critical behavioural demonstration to investigate whether face-selective regions forming the components of Haxby et al.'s (2000) core neural system for face perception are also sensitive to different aspects of the face image. To address this issue, we used contrast-reversal in combination with an fMR-adaptation paradigm (Andrews et al., 2010, Davies-Thompson et al., 2013, Harris et al., 2012) to determine the relative contribution of surface- and edge-based visual information to the neural representations underlying facial expression and identity. Importantly, for the fMR-adaptation paradigm we did not ask participants explicitly to attend to either expression or identity. Because previous studies have found that task can influence responses in regions of the ventral stream (Avidan et al., 2003, Ishai et al., 2004), our aim was to probe those aspects of the visual image coded by neural regions of interest irrespective of task. This was important because we did not want to bias the response in different regions with a task that could involve explicit or implicit judgements of either expression or identity. Instead, we used a neutral task of detecting a red spot positioned on some of the face images to ensure that participants looked at each face but did not need to respond to its identity or expression.

The fMR adaptation experiment investigated neural responses to stimulus blocks showing repeated images of the same face or a sequence that alternated between two different face images varying in identity and expression. The difference in overall response between blocks with repeated images and blocks with alternating different images gives a measure of neural adaptation. This measure of neural adaptation was applied to independently-localised face-selective regions thought to be involved in the perception of identity (FFA) and expression (pSTS) across three different image manipulation conditions. These conditions involved blocks in which stimuli were all contrast-positive (normal greyscale images), all contrast negative, or a mix of contrast-positive and contrast-negative images. Our hypothesis was that mixing normal and contrast-reversed images within a stimulus block should affect neural responses in face regions that are sensitive to surface-based cues, but it should not have a significant effect on responses in face regions that primarily represent edge-based information. Hence a region that shows adaptation to the blocks of mixed normal and contrast-reversed images must favour edge-based (shape) over surface-based (texture) information. This is a strong criterion because it involves adaptation to consistent shape cues present in normal and contrast-reversed images despite the substantial change in image properties.

## Materials and methods

### Participants

32 participants (21 females; mean age, 21) took part in the behavioural study (Experiment 1) and 25 different participants (16 females; mean age, 25 years) took part in the fMR-adaptation study (Experiment 2). All participants were right-handed and had normal or corrected-to-normal vision. All participants gave written informed consent. The study was approved by the YNIC Ethics Committee at the University of York.

### Stimuli

Face stimuli were Ekman faces selected from the Facial Expressions of Emotion Stimuli and Tests (FEEST) set (Young et al., 2002). The identities of these faces were unfamiliar to the participants. Four individuals posing five expressions (anger, disgust, fear, happiness and sadness) were selected based on the following three main criteria: (i) A high recognition rate for all expressions (mean recognition rate in a six-alternative forced-choice experiment: 94%; Young et al., 2002), (ii) consistency of the action units (muscle groups) across different individuals posing a particular expression, and (iii) visual similarity of the posed expression across individuals. Using these criteria to select the individuals from the FEEST set helped to minimise variations in how the expressions were posed. To avoid the use of the external features of the face which make little contribution to perception of expression, images were cropped so that only the internal features were visible. To generate the contrast-reversed faces, the value of each pixel in the image was subtracted from the mid-grey value and then added to the original grey value.

### Experiment 1

First, we determined the effect of contrast-reversal on perceptual judgements of facial identity and expression. Participants had to match the identity or expression of positive and negative faces. There were four stimulus conditions: (1) *same-expression*, *same-identity* (2) *same-expression*, *different-identity* (3) *different-expression*, *same-identity* and (4) *different-expression*, *different-identity*. Each trial consisted of 2 faces that were presented sequentially. Pairs of images were either both male or both female. Each face was presented for 900 ms and separated by an inter-stimulus interval of 300 ms. Trials were separated by 2.5 s during which participants had to report via a key press whether the identity or expression was the same/different (2AFC). Each phase of the experiment had two runs. In one run, participants matched expression, in the other identity. Both runs were identical in terms of the presented stimuli. The order of runs was counterbalanced across participants.

In the first phase of the experiment, images in each trial could be both positive or both negative. Each combination of contrast and condition was presented 20 times in a counterbalanced order, giving a total of 160 trials. 16 participants took part in the first phase of the experiment. In the second phase of the experiment, one image in each trial was positive and the other image was negative. The order of positive and negative images within trials was counterbalanced across conditions. Each condition was presented 32 times in a counterbalanced order, giving a total of 128 trials. 16 participants took part in the second phase of the experiment.

### Experiment 2

Next, we determined how face-selective regions in the brain (OFA, FFA, and pSTS) responded to blocks of positive, negative and mixed (positive and negative) contrast faces. To achieve this, the images used in Experiment 1 were incorporated into a block design fMR-adaptation paradigm in which stimuli were presented in blocks, with 6 images in each block. There were six conditions (types of block) in the experiment:

(1) *same-face*, *positive* — all 6 images in the block showed contrast-positive versions of the same face identity with the same expression
(2) *different-face*, *positive* — the 6 images in the block showed contrast-positive versions of two different face identities with two different expressions
(3) *same-face*, *negative* — all 6 images in the block showed contrast-negative versions of the same face identity with the same expression
(4) *different-face*, *negative* — the 6 images in the block showed contrast-negative versions of two different face identities with two different expressions
(5) *same-face*, *positive–negative* — the 6 images in the block showed alternating contrast-positive and contrast-negative versions of the same face identity with the same expression
(6) *different-face*, *positive*–*negative* — the 6 images in the block showed alternating contrast-positive and contrast-negative versions of two different face identities with two different expressions.

See Fig. 3A for examples of stimuli used in each type of block. For the ‘different-face’ blocks, the expression pairings were created in such a manner that each combination of expressions occurred equally often and the identity pairings were always faces of the same sex.

Within blocks, each face was presented for 900 ms and separated by a grey screen presented for 150 ms. The 900 ms presentation time was chosen to be the same as for the behavioural study (Experiment 1). Stimulus blocks were separated by a 9 s fixation grey screen. Each condition was presented 8 times in a counterbalanced order, giving a total of 48 blocks. Visual stimuli (6° × 8°) were presented 57 cm from participants' eyes in the scanner which was comparable to the visual angles used for the behavioural study. Images in the scanner were back-projected onto a screen within the bore. Participants were not asked to attend to identity or expression — only to view the images and to push a button when they detected a red dot, superimposed on 17% of the images. This red dot detection task also allowed us to monitor attention across stimulus blocks. No significant differences in red dot detection were evident across experimental conditions (Accuracy: 98%, F_(1,24)_ = .37; RT: 615 ms, F_(1,24)_ = .23).

This fMR-adaptation paradigm was used to determine the pattern of response to the different types of face images in different face regions (Grill-Spector and Malach, 2001, Grill-Spector et al., 1999). We were able to compare the response to the *same-face* and *different-face* condition across 3 image manipulations (*positive*, *negative* and *positive*–*negative* blocks). In the *same-face* conditions, one face image was repeated during the block. In the *different-face* conditions, 2 images with different identities and different expressions alternated during the block. In previous studies, we have found a release from adaptation to changes in identity in the FFA (Andrews et al., 2010, Davies-Thompson et al., 2013) and a release from adaptation to changes in expression in the pSTS (Harris et al., 2012). Because the *different face* conditions involve changes in both identity and expression compared to the *same face* conditions, regions involved in the processing of either facial identity or expression should show an increased response to the *different-face* compared to the *same-face* condition with *positive* contrast faces. However, if a region is sensitive to surface-based cues, we would expect that a release from adaptation would be attenuated by contrast reversal, particularly in the *positive*–*negative* conditions. On the other hand, if a region is only sensitive to edge-based cues, we would expect that adaptation should not be affected by contrast reversal, leading to adaptation even in the critical *positive*–*negative* conditions despite the substantial variations in overall image properties.

Face-selective regions were independently defined using a separate standard localiser scan (Harris et al., 2012, Kanwisher et al., 1997) in which the response to faces was compared to the response to objects, places and scrambled faces. Face images were taken from the Radboud Face Database (Langner et al., 2010) and varied in expression and identity. Images of objects and scenes came from a variety of internet sources. Images from each condition were presented in a blocked design with 5 images in each block. Each image was presented for 1 s followed by a 200 ms fixation cross. Blocks were separated by a 9 s grey screen. Each condition was repeated 5 times in a counterbalanced design. To ensure participants maintained attention throughout the experiment their task was to detect the presence of a red dot which was superimposed onto 20% of the images.

Imaging was performed using a GE 3T HD Excite MRI scanner at York Neuroimaging Centre at the University of York. A Magnex head-dedicated gradient insert coil was used in conjunction with a birdcage, radio-frequency coil tuned to 127.4 MHz. A gradient-echo EPI sequence was used to collect data from 38 axial slices (TR = 3, TE = 25 ms, FOV = 28 × 28 cm, matrix size = 128 × 128, slice thickness = 4 mm). Voxel size was 2.1875 × 2.1875 × 4 mm^3^. These were co-registered onto a T1-weighted anatomical image (1 × 1 × 1 mm) from each participant.

**Fig. 1:**
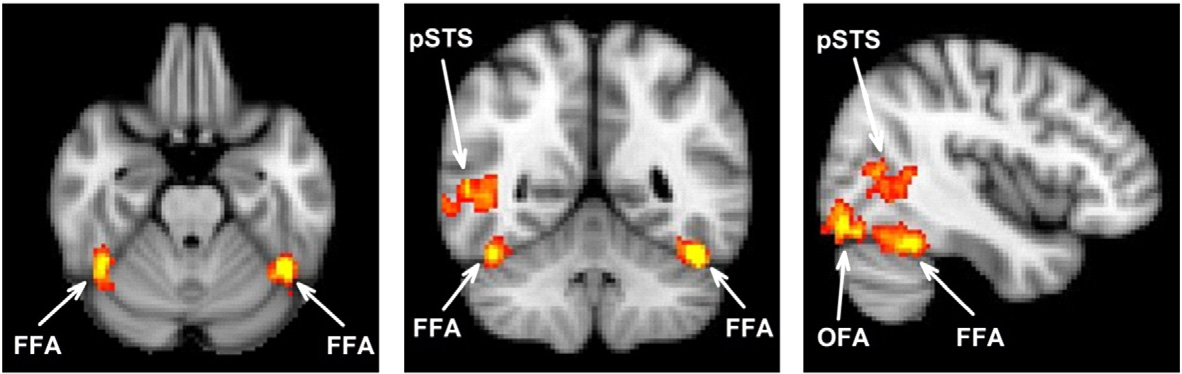
Average location of face selective regions in Experiment 2. Regions of interest were defined at the individual level from an independent functional localiser scan. Images are shown in radiological convention. The sagittal slice shows the right hemisphere.

The analysis of the fMRI data was performed using FEAT (http://www.fmrib.ox.ac.uk/fsl). The initial 9 s of data from each scan were removed to minimize the effects of magnetic saturation. Motion correction was followed by spatial smoothing (Gaussian, FWHM 6 mm) and temporal high-pass filtering (cutoff, 0.01 Hz). Face-selective regions were individually defined in each individual using the localiser scan by comparing the response to faces with the response to each non-face condition (object, place, scrambled face). These statistical maps were averaged and thresholded at p < 0.001 (uncorrected). Contiguous clusters of voxels located within the occipital and temporal lobes were defined as the FFA, OFA and pSTS in each participant (Table 1, Supplementary Table 1, Fig. 1).

**Table 1:** Mean MNI coordinates (mm) of the centre of gravity of face-selective regions across individuals. Face selective regions of interests were defined in individual EPI space and then transformed into MNI space. Standard error is reported in parenthesis.

Region	n	x	y	z	Volume (mm^3^)
FFA	23				
L		− 43 (0.6)	− 52 (0.8)	− 25 (0.4)	624.1 (117.8)
R		41 (0.7)	− 54 (1.4)	− 21 (0.7)	930.9 (117.5)
OFA	24				
L		− 43 (1.0)	− 79 (1.3)	− 11 (1.1)	784.4 (202.4)
R		44 (0.9)	− 81 (0.8)	− 14 (0.9)	1056.9 (208.3)
STS	19				
R		51 (1.0)	− 56 (2.2)	5 (1.0)	675.2 (225.3)

To analyse the data from the experimental scan, the time-course of response from each voxel was converted from units of image intensity to percentage signal change. All voxels within each ROI were then averaged to give a single time series for each ROI in each participant. Individual stimulus blocks were normalized by subtracting the response at every time point by the response at the start of the block and averaged to produce a mean time series for each condition for each participant. The normalized data were then averaged across participants to obtain the mean time-course for each stimulus condition. In order to determine significant differences in the peak response (average of 6 s and 9 s) to each stimulus condition repeated measures ANOVA were conducted across participants.

## Results

### Experiment 1

**Fig. 2:**
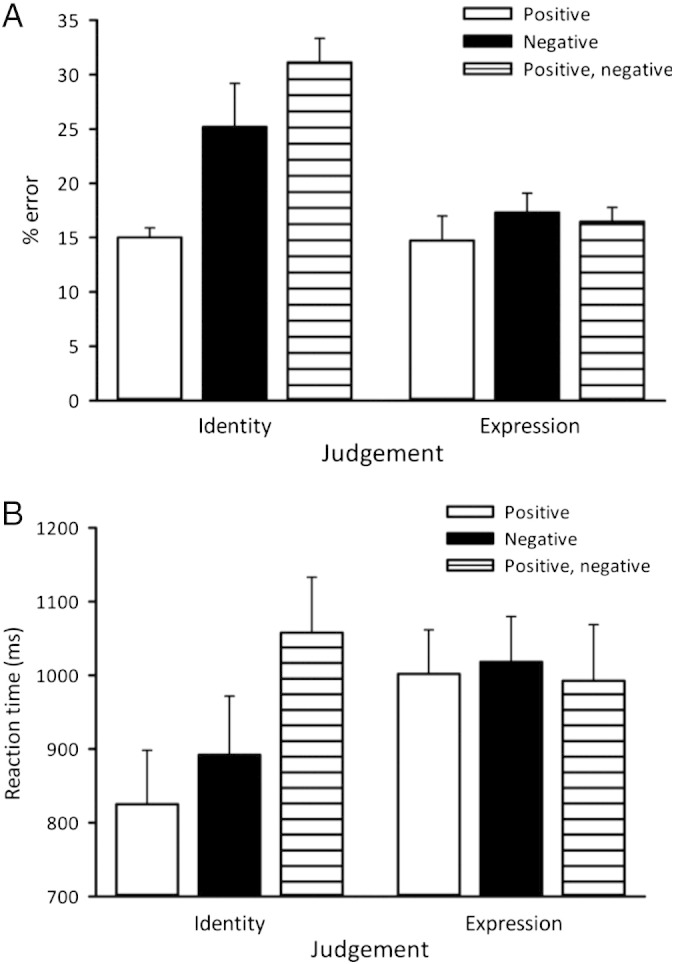
Experiment 1 — Behavioural discrimination of facial identity and expression. Images were presented in positive, negative, and mixed positive/negative formats. (A) Error rates and (B) reaction times significantly increased when judging facial identity but not facial expression in both negative and positive/negative conditions. Error bars represent SE. Note that the mixed positive/negative format is the most highly disruptive of identity judgements yet has no effect on expression judgements.

To determine the degree to which surface and edge information contribute to perceptual judgements of identity and expression, we compared performance with positive, negative, and mixed (positive and negative) contrast images. Participants were asked to report whether there had been a change in identity or a change in expression between successive images. Fig. 2 shows the % errors and reaction time during trials in which both images were positive, both images were negative or when one image was positive and the other negative. The results clearly show that there was a reduction in accuracy and an increase in mean reaction time (RT) on negative and on positive–negative trials compared to positive contrast trials for judgements of facial identity. In contrast, these manipulations had no effect on judgements of expression. To address the statistical significance of these differences, we compared % error and RT values for equivalent conditions on judgements of identity and expression. There was no difference in % error for judgements of identity and expression on positive contrast trials (t_(15)_ = 0.55, p = 0.59). However, there was a significant increase in % error for judgements of identity compared to expression on both negative (t_(15)_ = 2.81, p = 0.01) and positive/negative (t_(15)_ = 9.02, p < 0.001) trials. There was a higher RT for judgements of expression compared to judgements of identity on positive contrast trials (t_(15)_ = 3.73, p = 0.002). However, a difference between identity and expression was found for RT on negative (t_(15)_ = 2.46, p = 0.03) and positive–negative (t_(15)_ = 2.22, p = 0.04) trials.

### Experiment 2

To determine the effects of contrast reversal on the neural responses of regions previously linked to the processing of facial identity and expression, the images used in Experiment 1 were incorporated into an fMR-adaptation paradigm. Our objective was to determine how adaptation to faces was affected when images in the stimulus block were all positive, all negative, or alternated between positive and negative. A 2 × 2 × 3 × 2 ANOVA with the factors Region (FFA, OFA) Adaptation (same, different) Contrast (positive, negative, positive–negative) and Hemisphere (right, left) was conducted to determine whether the same regions of interest in the two hemispheres responded differently (Supplementary Fig. 1). The pSTS was not included in this part of the analysis as it was only identified in the right hemisphere. There was no main effect of Hemisphere (F_(1,10)_ < 1). The results also revealed no significant interaction between Contrast × Region × Adaptation × Hemisphere (F_(2,20)_ = 1.411, p = 0.27), Region × Hemisphere (F_(1,10)_ = .75), Adaptation × Hemisphere (F_(1,10)_ = 0.07), and Contrast × Hemisphere (F_(2,20)_ < 1). As there was no significant effect of hemisphere the timecourses were averaged across hemispheres resulting in three regions of interest; FFA, OFA, and right pSTS.

**Fig. 3:**
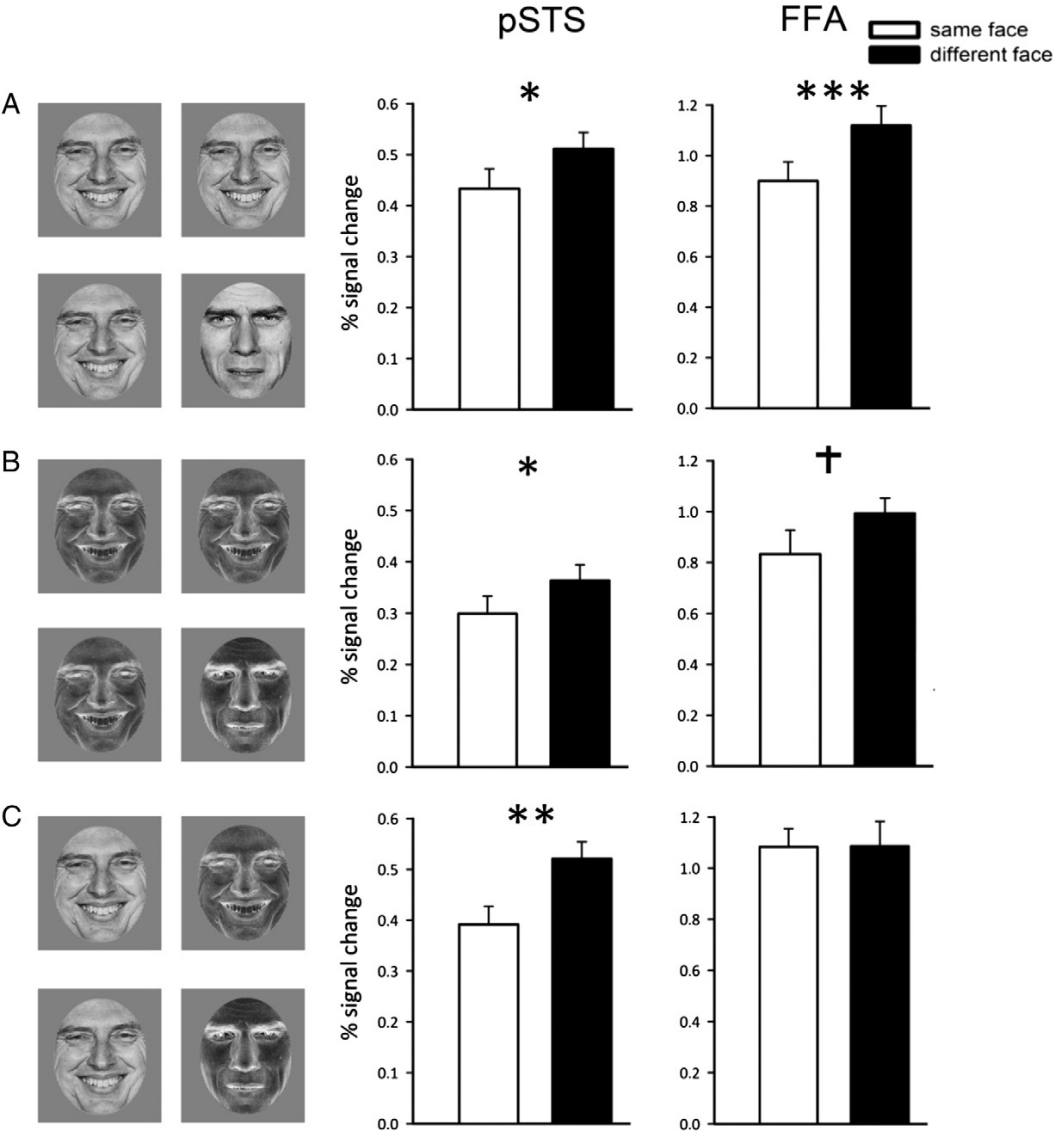
Experiment 2. (Left) Images in each stimulus block were from the same face or alternated between two different face images that varied in identity and expression. Same and different blocks were presented in (A) positive, (B) negative and (C) positive/negative contrast. (Right) Response in face-selective regions identified with an independent functional localiser scan to the same and different faces for each contrast condition. The results show that the pSTS was insensitive to the contrast polarity of the faces, showing a significantly bigger response to the different faces compared to the same faces across all contrast conditions. Conversely, a significantly bigger response to the different faces than same face blocks in the FFA was only evident when faces were in contrast positive. Critically, the pSTS shows adaptation even in the mixed positive–negative condition, whereas this completely eliminates adaptation in the FFA. † = p < 0.1,* = p < 0.05, ** = p < 0.01, ***p < 0.001.

The peak responses in each face-selective region (Fig. 3) were then analysed using a 3 × 2 × 3 ANOVA with Region (pSTS, FFA, OFA), Adaptation (same, different) and Contrast (positive, negative, positive–negative) as the factors. There were significant effects of Region (F_(2,30)_ = 35.40, p < 0.0001), Adaptation (F_(1,15)_ = 26.07, p < 0.0001) and Contrast (F_(2,30)_ = 8.242, p < 0.001). There was a significant interaction between Region × Contrast (F_(4,72)_ = 4.14, p < 0.05). There was no significant interaction between Region × Adaptation (F_(2,30)_ < 1), but there was as a significant interaction between Region × Adaptation × Contrast (F_(4,60)_ = 2.95, p < 0.05). To further investigate these effects, the responses in each face-selective region were investigated using a 3 × 2 ANOVA with Contrast (positive, negative, positive–negative) and Adaptation (same, different) as factors.

Fig. 3 shows the response in the pSTS. There was a significant effect of Contrast (F_(1,18)_ = 10.67, p < 0.005) and a significant effect of Adaptation (F_(1,18)_ = 10.36, p < 0.005). However, there was no significant Contrast × Adaptation interaction (F_(1,18)_ = 0.16), demonstrating that the degree of adaptation was not affected by changes in stimulus contrast. Adaptation was due to significantly bigger responses to the *different-face* conditions compared to the *same-face* conditions (*positive*: t_(18)_ = 2.64, p < 0.05, *negative*: t_(18)_ = 3.14, p < 0.01, *positive*–*negative*: t_(18)_ = 2.59, p < 0.05). The significant main effect of Contrast was driven by a bigger overall response to the *positive* contrast condition compared to the *negative* contrast condition (*same-face*: t_(18)_ = 2.54, p < 0.05; *different-face*: t_(18)_ = 3.44, p < 0.005).

In the FFA (see Fig. 3), there was a significant main effect of Contrast (F_(2,44)_ = 17.91, p < 0.0001) and Adaptation (F_(1,24)_ = 19.39, p < 0.0001). There was also a significant interaction between Contrast × Adaptation (F_(2,48)_ = 2.54, p < 0.05). This interaction reflected significantly larger responses to the *different-face* compared to the *same-face* condition for the *positive* condition (t_(22)_ = 6.09, p < 0.0001), a marginal, non-significant difference for the *negative* condition (t_(22)_ = 1.96, p = 0.06) and no difference in the *positive–negative* condition (t_(22)_ = 0.33). The significant main effect of Contrast was driven by a significantly bigger response to the *positive* compared to the *negative* contrast conditions for the *different-face* (t_(22)_ = 3.30, p = 0.003) but not the *same-face* (t_(22)_ = 1.40, p = 0.18) conditions.

The pattern of response in the OFA was similar to the FFA. There was a significant main effect of Adaptation (F_(1,23)_ = 13.82, p = 0.001) and Contrast (F_(2,46)_ = 11.20, p < 0.0001). There was also a significant interaction between Adaptation × Contrast (F_(2,46)_ = 3.35, p < 0.05). The interaction was due to differences between the *same-face* and *different-face* in the *positive* (t_(23)_ = 2.70, p < 0.05) and *negative* (t_(23)_ = 3.19, p < 0.005) but not in the *positive–negative* (t_(23)_ = 0.38) contrast conditions. The significant main effect of Contrast was driven by a significantly bigger response to the *positive* compared to the *negative* contrast conditions (*same-face*: t_(23)_ = 2.70, p < 0.01; *different-face*: t_(23)_ = 3.19, p < 0.005).

## Discussion

The aim of this study was to determine the contribution of image properties to the coding of faces in brain regions previously thought to be involved in the perception of facial identity and expression. To address this issue, we used contrast-reversal to reverse the pattern of light and dark across the image. This manipulation is critical because visual information conveyed by faces can be broadly organised into surface-based texture patterns and edge-based cues (Bruce and Young, 1998, Bruce and Young, 2012). Importantly, whilst contrast-reversal is highly disruptive of surface information, it does not affect the locations of image discontinuities (feature edges) critical to perceiving shape (Russell et al., 2006).

Behaviourally, we found that contrast-reversal affected perceptual judgements of facial identity, but not facial expression. In fact, expression could be judged as easily in mixed contrast pairs of images (one positive, one negative) as in consistent contrast pairs (both positive or both negative). This finding shows that the perceptual analysis of expression is largely insensitive to changes in surface textural properties of the image, since huge changes in these surface properties do not create any substantial interference with expression judgements.

From the fMR-adaptation data, a similar dissociation was evident in face-selective regions. The adaptation response in the FFA – a region thought to be linked with the perception of facial identity – was eliminated by mixing normal with contrast-reversed faces within a stimulus block. These findings are also consistent with single neuron recordings from neurons in face-selective regions of the macaque inferior temporal lobe, which show a robust selectivity for appropriate contrast relationships in face images (Ohayon et al., 2012). However, although our results show that the FFA is highly sensitive to surface-based cues, this need not imply that it is insensitive to shape-based cues. In a previous fMR-adaptation study, Jiang et al. (2009) used morphing techniques to generate faces that varied in either shape or surface texture and reported that the FFA was sensitive to changes in shape. Consistent with these results, other fMRI studies have shown that the FFA is sensitive to high-spatial frequency faces (Goffaux et al., 2011, Vuilleumier et al., 2003).

In marked contrast to the FFA, the adaptation response in pSTS – a region directly linked to the perception of expression – was unaffected by contrast-reversal, even when normal and contrast-reversed images were intermixed. Note that the fMR-adaptation task involved the detection of a red spot; participants were not asked to attend to differences in identity or expression. Hence differences between pSTS and FFA reflect differences in the perceptual coding mechanisms they use, not the task demands of attending to identity or to expression.

A key point arising from this pattern of results is to demonstrate how the apparent dissociations between the perception and neural representation of facial identity and expression might arise from image-based properties of faces rather than any inherent semantic distinction between identity and expression per se. Put simply, we have shown that the analysis of expression is strongly dependent on shape cues that convey changes from movements of the facial muscles and much less dependent on surface texture. None the less, it is clear that this is a relative dependence — for example, an open-mouthed smile introduces clear textural as well as shape changes in the mouth region. Likewise, behavioural work shows clearly that whilst surface texture is highly important to identity, shape also makes a contribution (Burton et al., 2005).

The apparent involvement of pSTS in expression perception and FFA in perception of identity may therefore reflect differential sensitivity to these properties of the facial image, in line with suggestions previously offered by Calder and Young (2005). This perspective concurs with image-based analyses of faces. Using PCA, Calder et al. (2001) found that Principal Components (PCs) that convey variation in shape were more effective in categorizing different facial expressions than PCs that convey variation in surface texture. Conversely, PCs that convey variation in surface texture were more effective for discriminating a change in identity or gender of a face compared to PCs that convey variation in shape.

It is interesting to consider, therefore, that the semantic categories of identity and expression might not necessarily be the underlying principle driving the functional organisation of these face-selective regions. Rather, the response in these regions may also reflect low-level properties inherent in the face image that are themselves relatively more informative for identity and expression. This is reflected in our fMR-adaptation results which show a dissociation between the processing of shape in the STS and surface information in the FFA. This different sensitivity to different image properties could give rise to the apparent dissociation between expression and identity. Consistent with this, Harris et al. (2012) found that the STS was sensitive to changes in shape-based facial features regardless of whether these changes altered the emotional category of the face. The functional architecture we propose seems plausible in the context of recent studies that have shown that more lateral and superior regions of the temporal lobe are sensitive to changes in the shape of objects, but more ventral and medial regions are more sensitive to the surface texture of objects (Cant and Goodale, 2007, Cant et al., 2009).

In conclusion, our results suggest that different visual information is used by brain regions thought to be involved in the processing of identity and expression. Regions involved in coding dynamic or changeable aspects of faces, important for the processing of expression, are relatively dependent on edge-based, shape information rather than surface-based cues. In contrast, regions involved in processing invariant aspects of faces, such as identity, are sensitive to surface-based as well as shape information. This offers an alternative perspective on the neural segregation of perception of identity and expression that puts image properties in the foreground.
