# Supplementary material for: Brain regions involved in processing facial identity and expression are differentially selective for surface and edge information
Source: Neuroimage. 2014 Aug 15;97:217–23. doi: 10.1016/j.neuroimage.2014.04.032 (PMC4077631; doi:10.1016/j.neuroimage.2014.04.032)
Supplement: Supplementary file 1 — Supplementary material. [file mmc1.pdf]

Psalta, L., Young, A.W., Thompson, P. and Andrews, T.J. (2013). The Thatcher illusion reveals orientation-dependence in brain regions involved in processing facial expression. *Psychological Science*, *24*, doi: 10.1177/0956797613501521.

Rhodes, G., & Tremewan, T. (1994). Understanding face recognition: Caricature effects, inversion and the homogeneity problem. *Visual Cognition, 1,* 275-311.

Rotshtein, P., Henson, R. N. A., Treves, A., Driver, J., & Dolan, R. J. (2005). Morphing Marilyn into Maggie dissociates physical and identity face representations in the brain. *Nature Neuroscience*, *8*, 107–113.

Russell, R., Sinha, P., Biederman, I., & Nederhouser, M. (2006). Is pigmentation important for face recognition? Evidence from contrast negation. *Perception*, *35*, 749–759.

Vuilleumier, P., Armony, J.L., Driver, J., Dolan, R.J. (2003). Distinct spatial frequency sensitivities for processing faces and emotional expressions. *Nature Neuroscience, 6,* 624-631.

Winston, J. S., Henson, R. N. A., Fine-Goulden, M. R., & Dolan, R. J. (2004). fMRI-adaptation reveals dissociable neural representations of identity and expression in face perception. *Journal of Neurophysiology*, *92*, 1830–1839.

White, M. (2001). Effect of photographic negation on matching the expressions and identities of faces. *Perception*, *30*, 969–981.

Young, A., Perrett, D., Calder, A., Sprengelmeyer, R., & Ekman, P. (2002). *Facial expressions of emotion - stimuli and tests (FEEST)*. Bury St Edmunds, England: Thames Valley Test Company.

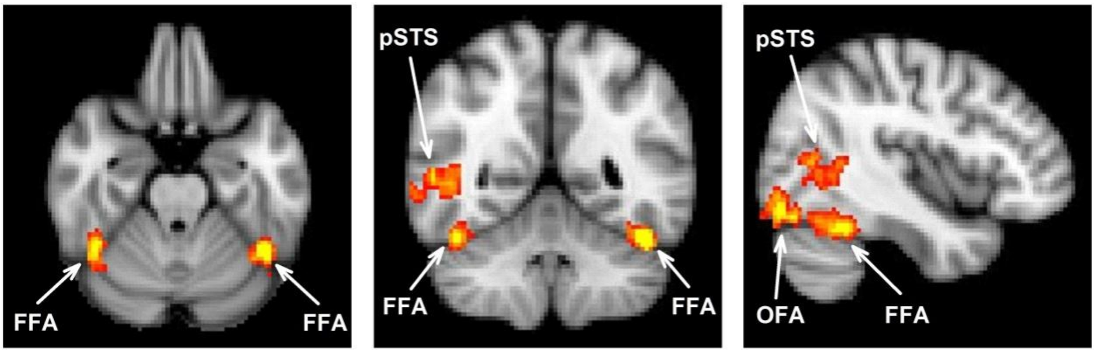

Figure 1 Average location of face selective regions in Experiment 2. Regions of interest were defined at the individual level from an independent functional localiser scan. Images are shown in radiological convention. The sagittal slice shows the right hemisphere.

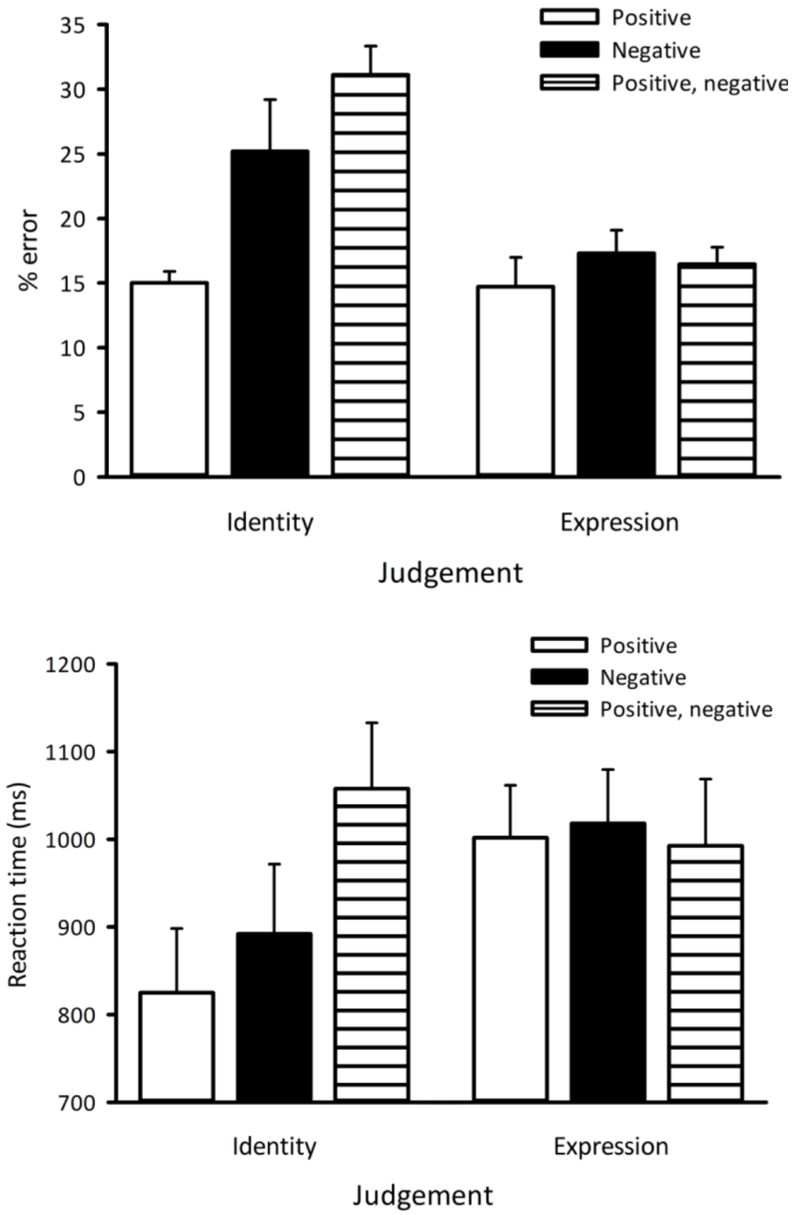

Figure 2 Experiment 1 - Behavioural discrimination of facial identity and expression. Images were presented in positive, negative, and mixed positive/negative formats. (a) Error rates and (b) reaction times significantly increased when judging facial identity but not facial expression in both negative and positive/negative conditions. Error bars represent SE. Note that the mixed positive/negative format is the most highly disruptive of identity judgements yet has no effect on expression judgements.
